# Improving Surgical Outcomes in Pancreatic Surgery With Preoperative Nutrition

**DOI:** 10.6004/jadpro.2014.5.2.4

**Published:** 2014-03-01

**Authors:** Dwanna Ward-Boahen, Meredith Wallace-Kazer

**Affiliations:** From St. Vincent’s Medical Center, Bridgeport, Connecticut, and Fairfield University School of Nursing, Fairfield, Connecticut

## Abstract

The purposes of this study were (1) to describe the relationship between preoperative physical status and postoperative outcomes in patients undergoing Whipple resection, and (2) to determine if the use of specialized immunonutrition with IMPACT Advanced Recovery supplementation improved postoperative outcomes (pancreatic leak rate, length of stay, and postoperative complications) in patients undergoing Whipple resection. The trial was a case-controlled prospective pilot study that took place in an outpatient gastrointestinal surgical oncology office in an urban community hospital in the northeast United States. The study population consisted of nine patients undergoing Whipple surgery. Patients were given IMPACT Advanced Recovery supplementation 4 days prior to Whipple surgery. Prospective data were collected on all patients and then compared to national averages in terms of outcomes. Study approval was obtained from the Fairfield University Institutional Review Board (IRB), though IRB approval was not required by the study facility due to the fact that this was a pilot study. Consent was also not required for retrospective chart review. Patients with lower scores according to the American Society of Anesthesiologists Physical Status Classification System have a shorter operating time in the setting of preoperative nutrition. Patients in this study who received preoperative nutrition with IMPACT Advanced Recovery supplementation had outcomes comparable to the national average. This pilot study suggests that there is a need for a multi-institutional randomized study powered to further evaluate the effectiveness of preoperative nutrition in pancreatic surgery. The literature supports the fact that preoperative nutritional supplementation should be offered to patients undergoing Whipple surgery. Optimization of nutritional status can translate to decreased length of stay and cost savings.

Pancreatic cancer is the fourth leading cause of cancer-associated death. Peak incidence is in the seventh and eighth decades of life, with a slightly higher incidence in African Americans vs. Caucasians. The incidence and mortality for pancreatic cancer has not changed over the past 2 decades (National Comprehensive Cancer Network [NCCN], 2014). Because symptoms are usually silent until the disease advances, over 50% of patients present with metastatic or locally advanced disease.

According to the NCCN (2014), risk factors associated with pancreatic cancer include smoking, increased body mass index, and occupational exposure to chemicals such as benzidine and beta-naphthylamine. Chronic pancreatitis has also been shown to result in up to a sevenfold increased incidence of pancreatic cancer. Diabetes is also a significant risk factor for pancreatic cancer. Pancreatic intraepithelial neoplasia is considered a precursor lesion along with intrapapillary mucinous tumors of the main pancreatic duct with elevated carcinoembryonic antigen levels. More recent studies also suggest that the consumption of processed meats is a significant risk factor (NCCN, 2014). Familial pancreatic cancer is rare; it is associated with germline mutation in CDKN2A (cyclin-dependent kinase inhibitor 2A) and breast cancer gene 2 (BRCA2).

Pancreatic cancer is a complex malignancy that requires management by a multidisciplinary team. Treatment options include chemotherapy, surgical resection, and/or radiation therapy. Neoadjuvant chemotherapy is not recommended in the setting of resectable disease unless performed under the auspices of a clinical trial. Complete surgical resection with negative margins gives the best chance for long-term survival (Morrison, 2010).

Over two-thirds of pancreatic cancers occur in the pancreatic head and are of ductal origin. The most common surgery for pancreatic head lesions is pancreaticoduodenectomy (commonly referred to as the Whipple procedure). This procedure is also used for patients with duodenal tumors, ampullary tumors, mucinous cystadenomas of the pancreas, neuroendocrine tumors, and cholangiocarcinomas. Whipple surgery involves removal of the antrum of the stomach, the head of the pancreas, the common bile duct, the gallbladder, the duodenum, and part of the jejunum.

Postoperative complications associated with the Whipple procedure are numerous and compounded by preoperative anorexia and malnutrition. This often leads to cancer-related cachexia (greater than 10% weight loss over a 6-month period). Factors that contribute to cachexia in cancer patients include a sustained proinflammatory cytokine response, poor dietary intake, and the catabolic effects of sepsis (Goonetilleke, Hathurusinghe, Burden, & Siriwardena, 2008). This underscores the importance of nutrition in patients with pancreatic cancer, especially those undergoing surgery. Weight stabilization is an important component in improving outcomes for patients after surgery. While postoperative nutrition has been extensively studied in multiple cancer types, the literature on preoperative nutrition is lacking.

This pilot study investigated whether physical status prior to surgical resection plays a role in outcomes. This study also investigated whether preoperative nutrition with enteral supplementation improves surgical outcomes in patients undergoing the Whipple procedure. Reducing postoperative complications has a large effect on outcomes. This includes decreasing length of hospital stay and postoperative infections and has a direct impact on cost savings.

## Background

Historically, there has been a high morbidity and mortality associated with the Whipple procedure; patients who are malnourished preoperatively have had worse outcomes. Over the past several decades, the procedure has improved. Mortality has decreased to less than 2% in high-volume surgery centers (Are, Dhir, & Ravipati, 2011). Overall, the mortality rate can vary from 1% to 12%. Schmidt et al. (2010) found that outcomes for patients undergoing the Whipple procedure are significantly improved in high-volume surgery centers with experienced surgeons. However, the morbidity rate has remained high.

Because a significant amount of the foregut (stomach, duodenum, gallbladder, and pancreas) is removed, major nutritional complications are associated with the Whipple procedure. These complications include delayed gastric emptying, pancreatic exocrine insufficiency, and diabetes (pancreatic endocrine insufficiency), which can lead to nutritional deficiencies (Pappas, Krzywda, & Mcdowell, 2010). Other complications include leaks at the anastomosis site, pancreatic fistula development leading to abscess formation, hemorrhage, biliary reflux, and marginal ulceration. A pylorus-preserving procedure was developed in an attempt to avoid some of these complications and also to improve operative time, blood loss, need for blood transfusion, and morbidity rate (Tran, 2009). However, studies have shown that there is no difference in the outcomes.

In an effort to improve outcomes postoperatively, preoperative nutrition has been recommended to have additional benefit, according to many studies. Drover and colleagues (2011) conducted a meta-analysis of 35 randomized controlled trials of patients receiving L-arginine–supplemented diets preoperatively to specifically look at the impact on length of stay and infectious complications. Most of the studies covered GI surgeries, though some focused on other types of procedures. There was a 41% reduction in infectious complications, and length of stay was decreased by 2 days. The conclusion was that all high-risk patients who undergo elective surgery and who have a high risk of infectious complications should have an L-arginine–supplemented diet. (L-arginine is a precursor for nitric oxide, which has been implicated in carcinogenesis. This mechanism in tumor biology is very complex, and further research is needed. There are no long-term studies that confirm adversarial use in nutritional supplementation.)

Senkal et al. (1999) conducted a randomized, double-blind study of 154 patients undergoing elective surgery for GI malignancies in which patients received 5 days of preoperative oral immunonutrition or an isoenergetic diet. Postoperatively, patients were given early immunonutrition with enteral feedings or an immune-modulated diet. It was found that immunonutrition with nutrients such as arginine, RNA, and omega-3 fatty acids decreased early occurrence of postoperative infection and substantially reduced costs associated with complications in upper GI surgery.

IMPACT Advanced Recovery (Nestle) is an oral nutritional supplement that contains L-arginine, omega-3 fatty acids, and nucleic acids. The synergistic effect of all three elements helps to modulate immune response after surgery. These amino acids, essential fatty acids, and enzymes are important in wound healing, dampening the inflammatory and immunosuppressive response, and activating special immune cells, respectively. Waitzberg et al. (2006) performed a meta-analysis of patients who received IMPACT as a part of their pre-, peri-, and/or postoperative management. Preoperative use of IMPACT was associated with an improvement in postoperative outcomes, leading to decreased length of stay and postoperative infectious complications. There was a 48% reduction in anastomotic leaks in GI surgical patients. IMPACT is indicated for use in many arenas, including the pre/postoperative settings, critical illnesses, burns, and wound management.

The purposes of this study were (1) to describe the relationship between preoperative physical status and postoperative outcomes in patients undergoing Whipple resection, and (2) to determine if the use of specialized immunonutrition with IMPACT improved postoperative outcomes (pancreatic leak rate, length of stay, and postoperative complications) in patients undergoing Whipple resection.

## Methods

A case-controlled, prospective pilot study was conducted in nine patients who had undergone Whipple resection for benign and malignant diseases of the pancreas from June 2010 to June 2011 at a high-volume surgery center (defined by the NCCN as performing more than 15 to 20 pancreatic resections annually). All surgeries were performed by a fellowship-trained hepatobiliary surgeon. Study approval was obtained from the Fairfield University Institutional Review Board (IRB), though IRB approval was not required by the study facility due to the fact that this was a pilot study. Consent was also not required for retrospective chart review.

Nine patients received 8 ounces of IMPACT Advanced Recovery supplementation twice a day for 4 days prior to elective Whipple surgery. The study group was compared to national averages in terms of three main outcomes: length of stay, presence and grade of leak, and postoperative complications. Additional information was gathered in terms of diagnosis, date of surgery, age, sex, comorbidities, blood loss during surgery, length of operation, and intensive care unit admission. Stage of malignancy was based on the American Joint Committee on Cancer (AJCC, 2012) guidelines (if applicable). The American Society of Anesthesiologists (ASA) classification system (1963) was used to compare morbidity and mortality by taking into account the patient’s preoperative physical status and surgical procedure complexity prior to selecting an anesthetic agent. The ASA scale ranges from 1 to 6. A score of 1 is given to patients who are healthy with no comorbidities. A score of 4 represents a patient who has major comorbidities and requires preoperative medical, cardiac, or pulmonary clearance.

## Data Analysis and Results

The study group was very similar to the pancreatic cancer population in terms of race, stage, and age. Nine patients underwent Whipple surgery and received IMPACT supplementation prior to their surgery. The mean age of the patients was 61 years (range, 49 to 85 years). Six of the nine patients were male; six patients were from minority groups (African American, Hispanic, Cape Verdean, and Jamaican), and three patients were Caucasian. A total of 88% of the patients had pancreatic adenocarcinomas, the majority of which were ductal in origin. Of the patients with a cancer diagnosis, 50% were stage IIB.

To determine if physical status/operative status was related to the outcomes of surgery, correlations were run between ASA grade, age, estimated blood loss, surgery time in minutes, and length of stay in days (Table 1). The average ASA score of the patients was 3. Only one patient had an ASA grade of 1 (healthy patient without comorbidities). There was no relationship between ASA grade, age, estimated blood loss, and length of stay. There was a positive relationship between ASA grade and operating room time measured in minutes (*p* = .001)

**Table 1:**
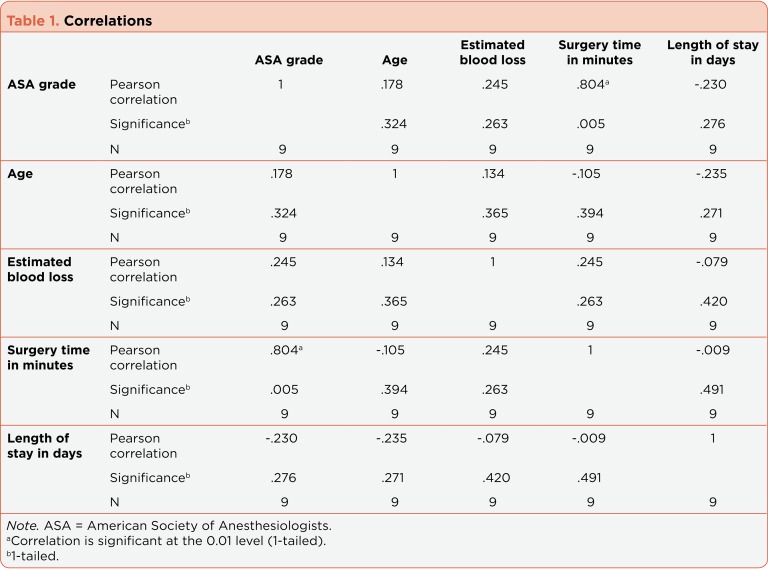
Table 1. Correlations

To determine if preoperative nutrition with IMPACT improved outcomes for patients undergoing Whipple resection, the study average was compared to national averages for several outcomes (Table 2). The data were then further analyzed, with an emphasis on patients with cancer.

**Table 2:**
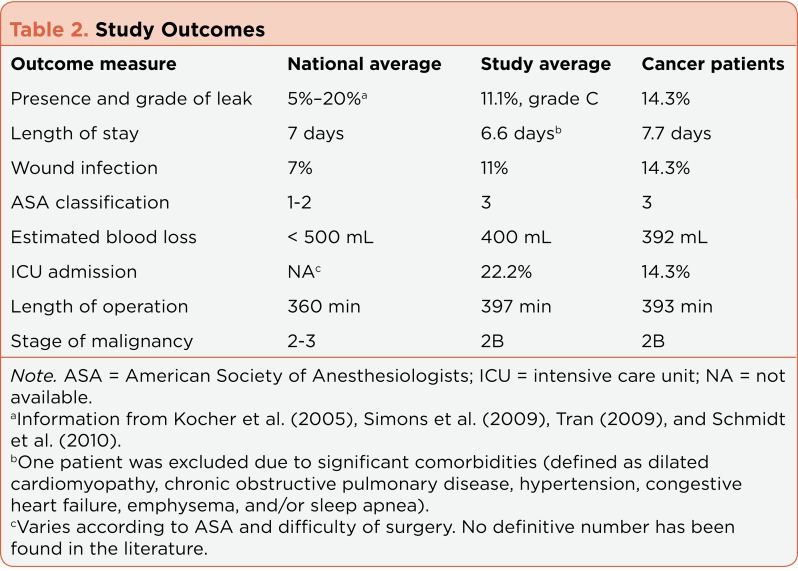
Table 2. Study Outcomes

## Discussion

Preoperative nutritional supplementation appears to be safe in pancreatic surgery, which falls under the umbrella of hepatobiliary surgery. Mann et al. (2010) performed an analysis to identify factors that affect outcomes in patients undergoing hepatobiliary surgery. Specifically looking at pancreatic cancer, factors that predicted adverse effects in the pre-, peri-, and postoperative settings included (1) age, (2) sex, (3) surgeon/hospital volume, (4) blood loss, (5) comorbidities, and (6) operative time. Most of the outcomes were analyzed within the study.

In this study, there was a trend toward improvement in outcomes with Whipple surgery when preoperative nutrition with IMPACT Advanced Recovery was given and analysis of some of the above-mentioned outcomes was undertaken. Though there was not a large change in outcomes when compared to the national average, the one factor that probably accounts for similar outcomes within all groups is the ASA score.

The ASA classification system was used in this study to assess preoperative physical status as a predictor of expected outcomes in the postoperative setting. The ASA grade of the study population was higher than the national average. This means that compared to the national average, the study population had more comorbidities. This may also account for the almost 30-minute longer operative time in the study group and a higher incidence of complications. In hepatobiliary surgery, 80% of patients have an ASA score of 1 to 2 (Kocher et al., 2005). Patients with lower ASA scores have shorter operating times. Patients with higher ASA scores would be expected to have longer operative times and complications perioperatively secondary to poorer physical status and multiple comorbidities.

It was also hypothesized that age, estimated blood loss, and length of stay would also have a relationship to ASA score, but these relationships were not statistically significant in the current study. Again, this relates back to the study population having higher ASA scores than the national pancreatic surgery population. Analyzing the data from the study, patients had similar outcomes with higher ASA scores than national results; this suggests that if ASA scores had been lower, a significant relationship would have been noted for outcomes with administration of IMPACT supplementation. An improvement in outcomes would have been noted, and this result would have shown a direction toward cost savings for the hospital.

Braga et al. (2002) conducted a cost-benefit analysis in patients undergoing GI surgery for cancer and found a cost savings secondary to avoidance of complications postoperatively. In their study, 305 patients were randomized to three groups: (1) preoperative nutrition with 5 days of IMPACT, (2) preoperative IMPACT along with jejunal feedings, or (3) no supplementation. When compared to the group with no supplementation, those who received preoperative nutrition consumed 78% of their diagnosis-related group (DRG) reimbursement rate, while the group with no supplementation consumed over 93% of their DRG. This underscores the fact that preoperative nutrition can have a significant impact on cost savings for the hospital.

Correlations were not run between stages of tumor, but it would be expected that an earlier stage tumor would allow for easier surgical resection (invasion of superior mesenteric artery, superior mesenteric vein, and/or portal vein place patients into borderline resectable or unresectable categories).

## Limitations

There were several limitations, including the setting of the study. It took place in a small community hospital and was based on a single surgeon’s experience with specialized training in hepatobiliary surgery. Another limitation was the study’s small sample size. A limited sample size (nine patients, in this case) may skew results. For example, only one patient had a wound infection, but the percentage was 14.3% of the study sample.

As it was not adequately powered, the study was performed as a pilot study. In order to have sufficient power, a multi-institutional randomized study needs to be conducted. There will need to be control for multiple surgical techniques, expertise, and institutional variability. A multi-institutional study would allow comparable control groups and the opportunity to study additional influences such as comorbidities and performance status.

Finally, length of supplementation may be considered a limitation to the study. IMPACT supplementation was administered to patients for 4 days vs. 5 days in the study by Waitzberg and colleagues, though the literature suggests that this time frame is adequate to modulate the immune-mediated response.

## Implications for Advanced Practice

Evaluation of nutritional status for patients with pancreatic cancer should include assessment of weight, diet, and pre-albumin. Screening tools such as the Subjective Global Assessment can be used. Preoperative nutritional supplementation prior to Whipple surgery, preferably with a product that has high levels of L-arginine, omega-3 fatty acids, and nucleotides, should be strongly considered.

Improvement in postoperative outcomes can translate into significant cost savings for the hospital. In addition, improved outcomes associated with pancreatic surgery can lead to early administration of adjuvant therapy and better toleration of these therapies. Advanced practitioners should be aware of the need for prompt early nutritional consults in this particular patient population.

## Future Directions

Risk assessment tools that can help to predict outcomes in hepatobiliary surgery are currently in development. These tools will help identify patients who are at increased risk for experiencing complications due to nutritional deficiencies and place an even more important emphasis on preoperative nutrition.

Other factors that were not analyzed in this study but that play a role in adverse outcomes associated with hepatobiliary surgery include low serum albumin, low serum creatinine, and cardiovascular disease. While this study focused on outcomes, examining these preoperative measures would be beneficial. This information is usually part of the preoperative workup prior to surgery, and the data would be easy to gather. The severity of these conditions, along with individual degree of cancer-related cachexia, may illuminate patients for whom preoperative nutrition would not be beneficial due to their inability to overcome these adverse factors.
